# Supplementary figures and images for: Disentangling the gut microbiota of Aldabra giant tortoises of different ages and environments
Source: PeerJ. 2025 Jun 10;13:e19566. doi: 10.7717/peerj.19566 (PMC12164811; doi:10.7717/peerj.19566)

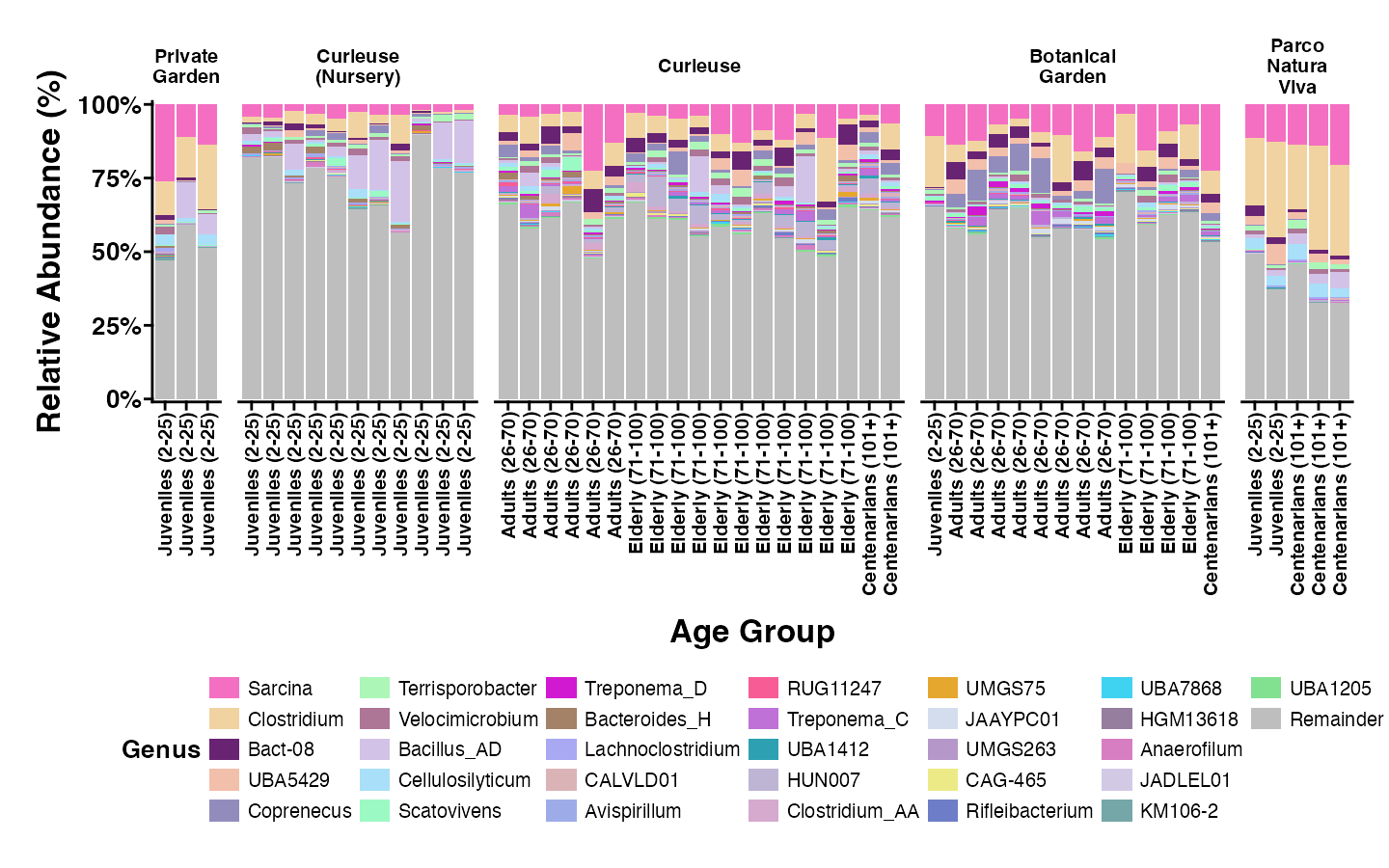

Supplement: Supplemental Information 4 — The samples are grouped by locations and are sorted by age group from left to right in each geolocational group. [file peerj-13-19566-s004.png]

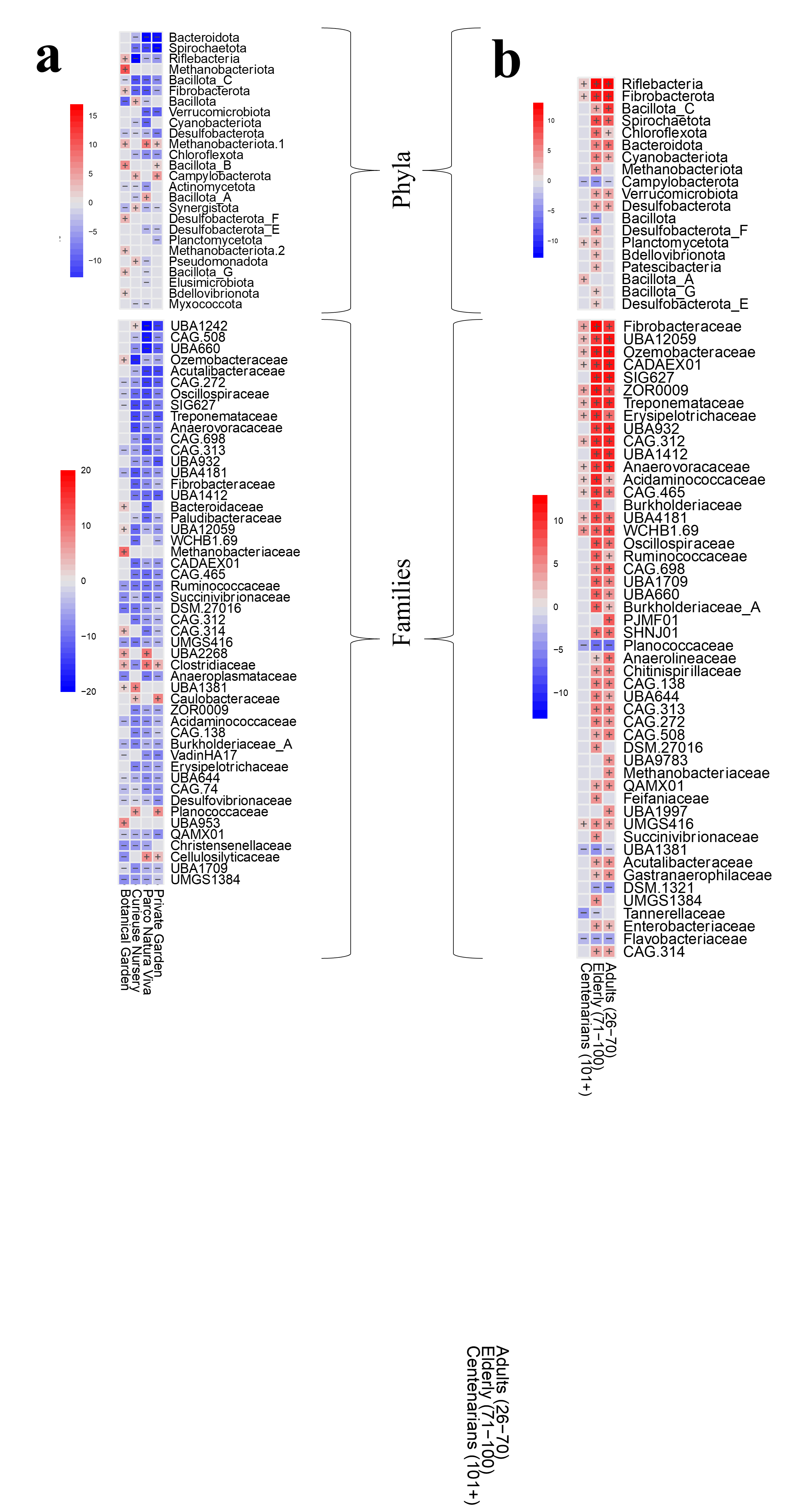

Supplement: Supplemental Information 5 — (A) Curieuse as a reference for environments. (B) Juveniles (2–25) as a reference for age groups. Values are for top 50 taxa with significant associations representing (–log(qval)*sign(coeff)). [file peerj-13-19566-s005.png]

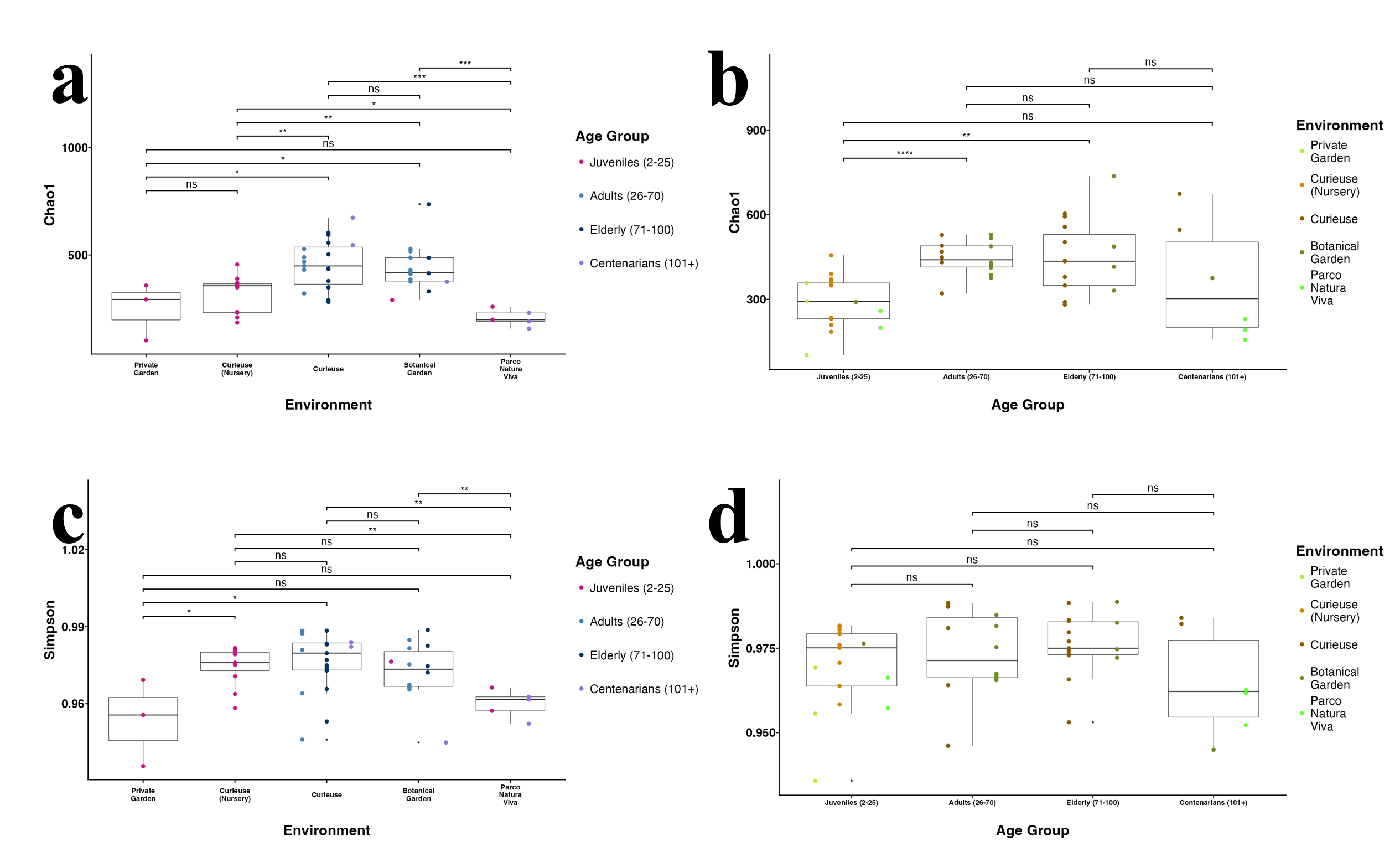

Supplement: Supplemental Information 6 — The pairwise Wilcoxon test was applied. [file peerj-13-19566-s006.png]

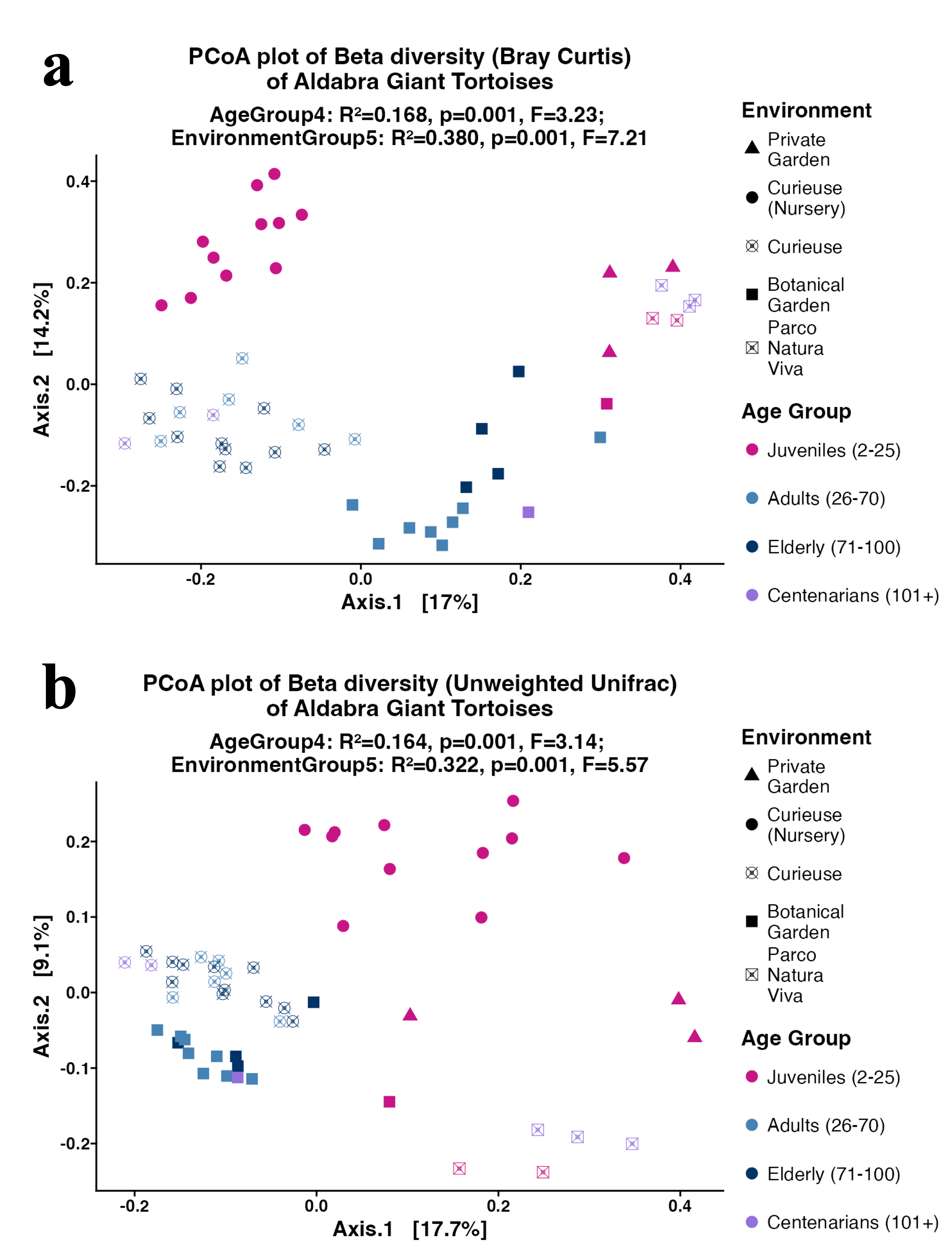

Supplement: Supplemental Information 7 — Sample colors indicate age groups and shapes indicate environments. The PERMANOVA test includes R-squared (R2), P-value, and pseudo F-statistics (F). [file peerj-13-19566-s007.png]
